# A196 CROHN’S DISEASE PATIENT DERIVED MACROPHAGES ARE MORE SUSCEPTIBLE TO HYDROGEN PEROXIDE INDUCED CELL DEATH

**DOI:** 10.1093/jcag/gwac036.196

**Published:** 2023-03-07

**Authors:** J Sousa, B Callejas, R Deshpande, M Yousuf, L Taylor, A Wang, D McKay, M Raman

**Affiliations:** 1 Department of Physiology & Pharmacology; 2 Department of Medicine, University of Calgary; 3 Snyder Institute for Crohnic Diseases; 4 Department of Community Health Sciences, University of Calgary, Calgary, Canada

## Abstract

**Background:**

Crohn’s disease (CD) is characterized by intestinal inflammation due to the interplay between immunity, genetics, and environmental factors such as diet. Selenium (Se) deficiency is common in patients with CD due to malabsorption or high enteric losses. Selenium is used in the synthesis of selenoproteins that have antioxidant properties (e.g. glutathione peroxidases (GPx)) and are highly expressed in macrophages. However, how Se deficiency affects immune system function in patients with CD is unknown. We hypothesize that characterizing Se status, selenoprotein expression and subsequently macrophage function will advance knowledge of mucosal immunity and provide novel insight into CD.

**Purpose:**

To determine if patients with active CD and healthy controls differ in Se dietary intake and status, oxidative stress, and macrophage cytotoxicity in response to oxidative stress.

**Method:**

Blood was collected from healthy volunteers and patients diagnosed with ileal, ileocolonic or colonic CD (age ≥18 years, with mild or moderate endoscopic disease activity or fecal calprotectin ≥250 µg/g, and Harvey Bradshaw index <16, stable medications including biologics for at least 8-weeks prior to recruitment). Serum was analyzed for GPx activity, malondialdehyde (MDA) and C-reactive protein (CRP) concentrations. Monocytes were isolated by plastic adherence and treated with M-CSF (10 ng/ml, 7d) to derive macrophages. mRNA expression of GPx1, GPx4 and SelenoP was determined by qPCR. Lactate dehydrogenase release was measured in macrophages treated with 500 µM H_2_O_2_ for 2h.

**Result(s):**

Samples and/or dietary intake data were collected from 9 patients with CD (3 female, 6 male, mean age=36.8 years) and 13 controls (7 female, 6 male, mean age=27.7 years). Dietary Se intake did not differ between patients with CD and controls (126.1 ± 23.2 vs. 123.3 ± 19.8 µg/day). GPx activity was greater in the serum of patients with CD compared to controls (369 ± 49 vs. 169 ± 27 mU/mL, n=6-8, p<0.005). Patients with CD and controls did not differ in serum MDA concentration (7.80 ± 0.57 vs. 6.53 ± 1.1 µM). CRP levels correlated with serum MDA concentration in patients with CD (r=0.95, n=5, p<0.05) but not GPx activity. Macrophages from patients with CD (n=6) and controls (n=7) did not differ in expression of GPx1 and GPx4 mRNA, whereas SelenoP mRNA was ~200-fold lower in macrophages from patients with CD. Macrophages derived from patients with CD were more susceptible to H_2_O_2_-evoked cell death (10.3 ± 1.1 vs. 4.7 ± 0.7 % n=2-3 p<0.05).

**Conclusion(s):**

Despite adequate dietary Se intake our findings suggest altered Se metabolism in patients with active CD, with increases in serum GPx potentially indicative of the need for antioxidant activity to counter oxidative stress. The increased sensitivity of macrophages from patients with CD to H_2_O_2_ emphasizes the role of oxidative stress and redox balance in IBD. Defining how micronutrients, in this instance Se, impacts innate immunity may provide new approaches to the management of CD.

**Disclosure of Interest:**

None Declared

